# Effect of galectin-3 on the behavior of Eca-109 human esophageal cancer cells

**DOI:** 10.3892/mmr.2014.2873

**Published:** 2014-11-05

**Authors:** NING LIANG, XIAOMING SONG, JIAN XIE, DEGUO XU, FENGJUN LIU, XINSHUANG YU, YUAN TIAN, ZHEN LIU, LILI QIAO, JIANDONG ZHANG

**Affiliations:** 1 Division of Oncology, Department of Graduate, Weifang Medical College, Jinan, Shandong 261053, P.R. China; 2 Department of Thoracic Surgery, Qianfoshan Hospital Affiliated to Shandong University, Jinan, Shandong 250014, P.R. China; 3 Department of Radiation Oncology, Qianfoshan Hospital Affiliated to Shandong University, Jinan, Shandong 250014, P.R. China

**Keywords:** esophageal carcinoma, galectin-3, Eca-109 cells

## Abstract

Galectin-3, a β-galactoside-binding lectin, is a cell adhesion molecule involved in the regulation of tumor progression. However, the importance of galectin-3 in Eca-109 human esophageal cancer cells has not yet been elucidated. In the present study, a lentiviral vector was designed for overexpression of galectin-3 in Eca-109 cells following plasmid-mediated transfection (Eca-109/Gal-3 cells). A negative lentiviral vector was introduced into Eca-109 cells as a control (Eca-109/Neo cells). Western blot and reverse transcription-polymerase chain reaction analyses were used to measure the expression levels of galectin-3 protein and mRNA. The proliferation of Eca-109 cells was measured by a cell counting kit-8 assay. Eca-109 cell apoptosis was determined by Annexin V/7-amino-actinomycin double-staining. The migration and invasion capacity of Eca-109 cells was determined by a Transwell assay. A total of >98% Eca-109 cells were transfected with the lentiviral vector harboring galectin-3, and galectin-3 expression was detected in Eca-109 cells, Eca-109/Gal-3 cells and Eca-109/Neo cells. Compared with non-transfected and negative control Eca-109 cells, proliferation was increased significantly in the Eca-109/Gal-3 cells (P<0.05). Galectin-3 also significantly reduced Eca-109 cell apoptosis, compared with the two control groups (P=0.007 and P=0.04, respectively). Transwell migration and invasion assays revealed that significantly greater numbers of Eca-109/Gal-3 cells crossed the artificial basement membrane (55.4±3.9) compared with either the non-transfected or negative control Eca-109 cells (30.6±1.5 and 29±2.6 respectively, P<0.05). In conclusion, galectin-3 expression was significantly increased in transfected Eca-109 esophageal cancer cells, resulting in enhanced proliferation, migration and invasion, as well as reduced apoptosis. These data indicate that galectin-3 may be a potential molecular target in the treatment of esophageal cancer.

## Introduction

Esophageal cancer is an aggressive cancer with an annual mortality rate almost matching incidence. At diagnosis, 54% patients present with either localized or regional disease (1). At the early stages of tumor development, a variety of changes in cell-cell and cell-matrix interactions result in abnormal cell behavior, which may induce invasion and malignant transformation. Resistance to apoptosis enables tumor cells to escape the effects of anticancer drugs and natural effector cells, rendering effective treatment difficult. Furthermore, the characteristics of this type of tumor contribute to disease progression and increased malignancy. During the late stages of tumor development, metastasis is the predominant complication and renders this type of tumor difficult to control. Therefore, elucidation of the mechanism underlying disease progression and metastasis is urgently required.

Galectin-3, a β-galactoside-binding lectin, which belongs to a widely distributed galectin family, contains a carboxyl-terminal carbohydrate recognition domain with an amino-terminal tandem repeat (2). The protein has an extended N-terminal tail consisting of a single polypeptide containing 8–13 consensus 9-mer amino acid repeats rich in proline, tyrosine and glycine (3). In the past decade, galectin-3 has been demonstrated to be widely expressed in tumor cells (4,5), and galectin-3 expression has been shown to be involved in various biological phenomena, including cell growth, adhesion, differentiation, apoptosis, cancer aggressiveness and metastasis (6,7). Recently, galectin-3 expression levels have been demonstrated to be correlated with neoplastic transformation in certain malignancies (6). Schoeppner *et al* (8) observed significantly increased galectin-3 expression levels in high-grade dysplasia and early invasive colon carcinoma, which exhibited a linear association with advancing stage. Canesin *et al* (9) demonstrated that galectin-3 expression contributed to disease progression and poor survival in advanced bladder cancer patients. A study by Sakaki *et al* (10) indicated that the overexpression of galectin-3 in clear cell renal cell carcinoma is a predictor of disease progression and metastasis. Knapp *et al* (11) demonstrated that galectin-3 localization in benign, adjacent-benign and tumor tissues was significantly correlated with biochemical recurrence in prostate specimens. Braeuer *et al* (12) observed that increased galectin-3 expression levels in melanoma exerted profound effects on tumor growth and metastasis. However, galectin-3 expression has been revealed to be downregulated in other types of malignancy, including breast, ovarian and uterine carcinomas (7,13).

The effects of galectin-3 expression on tumor progression and metastasis in esophageal cancer, which is an aggressive malignancy, have not yet, to the best of our knowledge, been clarified. Therefore, in the present study, the effect of galectin-3 on the behavior of the Eca-109 esophageal cancer cell line was investigated.

## Materials and methods

### Eca-109 cell culture

Eca-109 human esophageal cancer cells were obtained from Shandong Academy of Medical Sciences (Shandong, China). The cells were incubated at 37°C in 5% CO_2_ in plastic tissue culture flasks (Corning Inc., Acton, MA, USA) with complete Dulbecco’s modified Eagle’s medium (DMEM)-F12 (Gibco-BRL, Carlsbad, CA, USA) containing 10% fetal bovine serum (FBS; Gibco-BRL) and 1% penicillin-streptomycin (HyClone; Thermo Fisher Scientific, Rockford, IL, USA). Cobble-shaped cells began to expand after two days. At ~80% confluence, the cells were separated by digestion with 0.25% trypsin-EDTA (Gibco-BRL) and passaged into three plastic tissue culture flasks in the growth medium for expansion.

### Galectin-3 lentiviral vector generation

**Figure 1:**
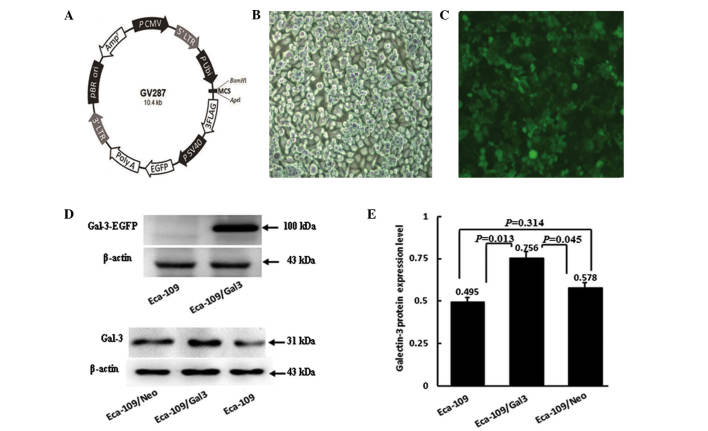
Enhanced green fluorescent protein (EGFP) + galectin-3 (Gal-3) immunosignals (100 kDa) were detected in the Eca-109/Gal-3 cells but not in Eca-109 cells without galectin-3 transfection. (A) GV287 plasmid carrier, AgI cleavage. (B) Non-transfected Eca-109 human esophageal cancer cells. (C) (Gal-3)-transfected Eca-109 cells exhibited transfection efficiency >95%. (D and E) Western blot analysis of Gal-3 expression levels in Eca-109, Eca-109/Gal-3 and Eca-109/Neo cells. Gal-3 immunosignals (31 kDa) were detected in Eca-109, Eca-109/Gal-3 and Eca-109/Neo cells. However, signal densities were significantly stronger in Eca-109/Gal-3 cells than in either Eca-109 or Eca-109/Neo cells (P=0.013 and P=0.045, respectively). No significant differences in signal density were detected between Eca-109 and Eca-109/Neo cells (P=0.314).

The following galectin-3 gene sequence: 5′-CAGGAGAGTCATTGTTTGCAA-3′, with a G/C content of 42.1%, was obtained from GenBank (Accession no. NM_02306). Shanghai Ji Kaiji Chemical Technology Co., Ltd. (Shanghai, China) designed the GV287 AgI cleavage lentivirus recombinant target gene plasmid containing enhanced green fluorescent protein (EGFP). This viral vector frame sequences were designed by Shanghai Ji Kaiji Chemical Technology Co., Ltd as viral vector for overexpression of galectin-3 in Eca-109 cells. The viral vector frame sequences were as follows: 5′-TCAGGAGAGTCATTGTTTGCAATTCAAGAGATTGCAAACAATGACTCTCCTGTTTTTTC-3′, 5′-TCGAGA AAAAACAGGAGAGTCATTGTTTGCAATCTCTTGAAT TGCAAACAATGACTCTCCTGA-3′. The target gene segment was ligated into the GV287 vector (Fig. 1A; Shanghai Ji Kaiji Chemical Technology Co., Ltd.).

### Lentiviral transfection of Ecal09 cells

Eca-109 cells were transfected with the lentiviral vector according to the lentiviral vector particle end-user operation manual provided by Shanghai Ji Kaiji Chemical Technology Co., Ltd. Preliminary transfection experiments were conducted to confirm the optimal concentration of lentivirus; transfection reagent was provided by the same company. At 80% confluence, the Eca-109 cells were released into a single cell suspension by digestion with trypsin-EDTA and seeded at 3,000–5,000 cells/ml) in 96-well tissue culture plates (Corning Inc.). After 24 h, the cells were inoculated with four concentrations of lentivirus (multiplicity of infection = 10, 20, 50 and 100, respectively) and incubated at 37°C in 5% CO_2_. After 8 h, the transfection medium was replaced with complete growth DMEM. After three days, the optimal conditions for transfection were determined according to the intensity of green fluorescent protein (GFP) expression evaluated using an inverted fluorescence microscope (FSX100, Olympus Corporation, Tokyo, Japan). On this basis, Eca-109 cells were seeded (30,000–50,000 cells/ml) in 6-well tissue culture plates (Corning Inc.). At 70–80% confluence, the cells were digested and passaged into 25-cm^2^ cell culture flasks in growth medium (complete DMEM -F12 containing 10% FBS) for expansion. In addition, virus without anti-Smad was used to transfect Eca-109 cells, serving as a negative control (Eca-109/Neo). The recombinant galectin-3 lentiviral vector-transfected Eca-109 cells were designated Eca-109/Gal-3.

### Western blot analysis

Total protein extracts from Eca-109, Eca-109/Neo and Eca-109/Gal-3 cells were homogenized with radioimmunoprecipitation assay lysis solution (Sigma-Aldrich, St. Louis, MO, USA) and centrifuged at 12,000 × g for 30 min at 4°C. The supernatant was collected following centrifugation and protein concentrations were determined using a Bicinchoninic Acid Protein Assay kit (Pierce Biotechnology, Inc., Rockford, IL, USA). Total protein extracts were separated by 10% sodium dodecyl sulfate-polyacrylamide gel electrophoresis (SDS-PAGE) and transferred to a polyvinylidene membrane (Millipore, Billerica, MA, USA). Western blot analysis was conducted by incubating the membrane with monoclonal mouse anti-galectin-3 antibody containing a recombinant fragment of human galectin-3 full-length protein (1:1,000; Abcam, Cambridge, UK) overnight at 4°C. The membranes were subsequently rinsed with wash solution and incubated with sheep anti-rat IgG conjugated to peroxidase (1:500; Sigma-Aldrich). Immunosignals were visualized with the Protein Detector 5-bromo-4-chloro-3-indolyl-phosphate/nitro blue tetrazolium western blotting kit (Beyotime Biotech., Jiangsu, China) according to the manufacturer’s instructions. Enhanced chemiluminescence (ECL; Millipore Corporation, Billerica, MA, USA) images were captured with a FluorChem E instrument (Cell Biosciences, Santa Clara, CA, USA). The quantification of each sample was conducted using ImageQuant 5.2 software (GE Healthcare, Little Chalfont, UK). A separate membrane was prepared using the same methods, and was probed with mouse monoclonal anti-β-actin (1:1,000; Santa Cruz Biotechnology, Inc., Santa Cruz, CA, USA) and mouse monoclonal anti-GFP (1:1,000; CoWin Biotech Co., Ltd., Beijing, China) antibodies.

### Quantitative polymerase chain reaction (qPCR) analysis

Reverse transcription-PCR and qPCR were used for galectin-3 gene expression analysis. Total RNA was isolated from cultured Eca-109 and Eca-109/Gal-3 cells using RNA-solv reagent (Omega Bio-Tek, Norcross, GA, USA) according to the manufacturer’s instructions. The total RNA concentration was determined by spectrophotometry (SPECTRA MAX190; Molecular Devices, Sunnyvale, CA, USA). Complementary DNA (cDNA) was synthesized from 1 μg total RNA using a Rever Tra Ace^®^ qPCR-RT kit (Toyobo, Osaka Japan) according to the manufacturer’s instructions.

PCR reactions were conducted using an ABI ViiA7 Dx instrument (Life Technologies, Waltham, MA, USA) following the manufacturer’s instructions, and performed in a 10 μl reaction mixture containing 1 μl cDNA, 5 μl SYBR^®^ Green (Toyobo), 1 μl of each primer and 2 μl H_2_O. The reaction conditions were as follows: 10 sec at 65°C, followed by 60 cycles of 5 sec at 60°C and 10 sec at 72°C, then 30 sec at 65°C. Gene-specific primers were designed as determined by the following human galectin-3 mRNA sequences in GenBank (Accession number, NM_02306): Forward: 5′-GGTGAAGCCCAATGCAAACA-3′ and reverse: 5′-TGCAACCTTGAAGTGGTCAG-3′.

Amplification of human β-actin mRNA served as a reference to normalize sample loading using the following primers: Forward, 5′-TGGCACCCAGCACAATGAA-3′ and reverse: 5′-CTAAGTCATAGTCCGCCTAGAAGCA-3′.

PCR results were quantified using the ΔCt method according to the following formula: Ratio = 2-ΔCt, where ΔCt = Ct target gene − Ct endogenous control gene (β-actin) (14).

### Cell proliferation assay

Eca-109, Eca-109/Neo and Eca-109/Gal-3 cell suspensions (100 μl) were dispensed into 96-well round-bottomed microtiter plates (3,000 cells/well) and incubated for 12–72 h at 37°C in 5% CO_2_. Subsequently, 10 μl cell counting kit-8 (CCK-8; Dojindo, Kunamoto, Japan) solution was added to each well and the cells were incubated for a further 4 h. The absorbance was measured at 450 nm with a spectrophotometer (Spectramax 190; Molecular Devices, Sunnyvale, CA, USA). Growth curves were generated from the average values of five wells in each group.

### Cell apoptosis assay

Cell apoptosis was analyzed by flow cytometry (FACSAria II; BD Biosciences, Franklin Lakes, NJ, USA). Cultured cells were washed twice with phosphate-buffered saline (PBS) and resuspended in binding buffer at 1×10^6^ cells/ml. Cell suspensions (1×10^5^ cells/100 μl) were transferred to 5 ml culture tubes, and 5 μl Annexin V-phycoerythrin (PE; eBioscience, San Diego, CA, USA) and 5 μl 7-amino-actinomycin (7-AAD; eBioscience) were then added. The cells were gently vortexed and incubated at room temperature in the dark for 15 min. Subsequently, another 400 μl binding buffer was added. Flow cytometry was performed within 4 h staining.

### Transwell migration assay

Transwell insert (8.0 μm pore size) polycarbonate filters (Costar^®^; Sigma-Aldrich) were used to examine the effect of galectin-3 on cell migration. Serum-free medium single-cell suspensions (5×10^4^ cells/200 μl) were added into the upper chamber and 500 μl complete DMEM medium was added to the lower chamber. Following incubation for 24 h, the filters were immersed in methanol for 15 min at room temperature, then with 0.25% crystal violet stain for 10 min at room temperature prior to washing with water. The cells that had migrated to the lower side of the filter were counted with an inverted fluorescence microscope.

### Matrigel invasion assay

The Transwell insert (8.0 μm pore size) polycarbonate filters were used to investigate cell invasion. The upper chamber was precoated with 100 μl 1:5 diluted Matrigel (Becton-Dickinson, Franklin Lakes, NJ, USA). Serum-free medium single-cell suspensions (1×10^5^ cells/200 μl) were added to the upper compartment of the precoated units. The units were then transferred to wells containing 500 μl complete DMEM medium and incubated for 48 h. The cells and Matrigel on the upper surface of the membrane were removed with a cotton bud, then the membrane was washed with PBS three times, immersed in methanol at room temperature for 15 min and then immersed in 0.1% crystal violet stain for 10 min at room temperature prior to washing with water. The cells that had migrated through the pores to the lower side were counted with an inverted fluorescence microscope.

### Statistical analysis

All data were analyzed with SPSS 13.0 (SPSS, Inc., Chicago, IL, USA). The data are presented as the mean ± standard deviation. Unpaired Student’s t-tests were performed for comparisons between two groups. P<0.05 was considered to indicate a statistically significant difference.

## Results

### Galectin-3 gene transfection

Preliminary experiments investigating the transfection of Eca-109 cells showed that >98% the cells were found to emit green fluorescence following transfection (Fig. 1B and C), indicating stable expression of the galectin-3 gene.

### Galectin-3 expression levels in Eca-109 cells

The levels of galectin-3 protein expression were detected in Eca-109, Eca-109/Neo and Eca-109/Gal-3 cells by western blotting. A 31-kDa band was detected in the Eca-109/Gal-3 group as well as in the Eca-109 and Eca-109/Neo control groups. EGFP + galectin-3 protein (100 kDa) was detected in Eca-109/Gal-3 cells but not in Eca-109 cells (Fig. 1D). The protein expression levels were significantly higher in the Eca-109/Gal-3 group than in the Eca-109 and Eca-109/Neo control groups (P=0.013 and P=0.045, respectively), but no significant differences were detected between the Eca-109 and Eca-109/Neo group protein levels (P=0.314; Fig. 1E).

The expression levels of galectin-3 mRNA in the Eca-109 cancer cells were quantified by qPCR. Galectin-3 mRNA was detected in Eca-109, Eca-109/Neo and Eca-109/Gal-3 cells, indicating that the galectin-3 is endogenously expressed in Eca-109 esophageal cancer cells. Compared with Eca-109 and Eca-109/Neo cells, galectin-3 mRNA expression levels in Eca-109/Gal-3 cells were increased 1.13- and 1.36-fold, respectively (P<0.05). No significant differences were identified between the Eca-109 and Eca-109/Neo cell galectin-3 mRNA expression levels (P>0.05).

### Effect of galectin-3 on the proliferation of Eca-109 cells

**Figure 2:**
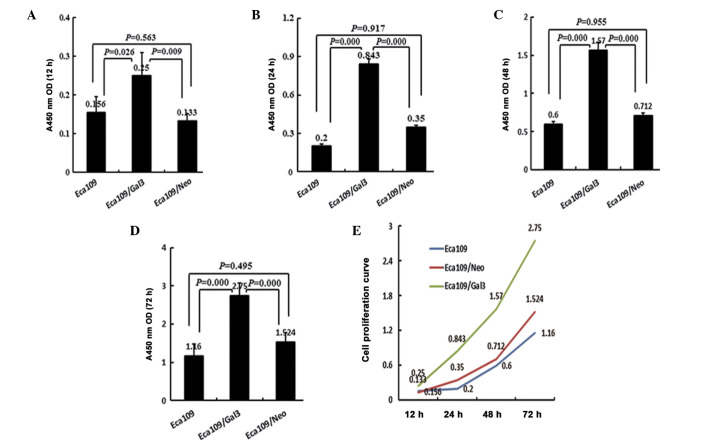
Effect of galectin-3 overexpression on the proliferation of Eca-109 human esophageal cancer cells was determined by a cell counting kit-8 assay. After (A) 12, (B) 24, (C) 48 and (D) 72 h culture, the *in vitro* growth of the Eca-109/Gal-3 group was significantly increased compared with that of either the Eca-109 or the Eca-109/Neo group (P<0.05). However, no significant differences were identified between the Eca-109 and Eca-109/Neo groups (P>0.05). (E) Growth curves of Eca-109 cells in the three groups.

The effect of galectin-3 overexpression on the proliferation of Eca-109 cancer cells was determined by the CCK-8 assay. Subsequent to culture for 12, 24, 48 and 72 h, the *in vitro* growth of the Eca-109/Gal-3 group was significantly higher than that of either the Eca-109 or Eca-109/Neo groups at all time points (P<0.05). No significant differences were detected between the Eca-109 and Eca-109/Neo groups at time point (12 h, P=0.563; 24 h, P=0.917; 48 h, P=0.955; 72 h, P=0.495; Fig. 2).

### Effect of galectin-3 on Eca-109 cell apoptosis

**Figure 3:**
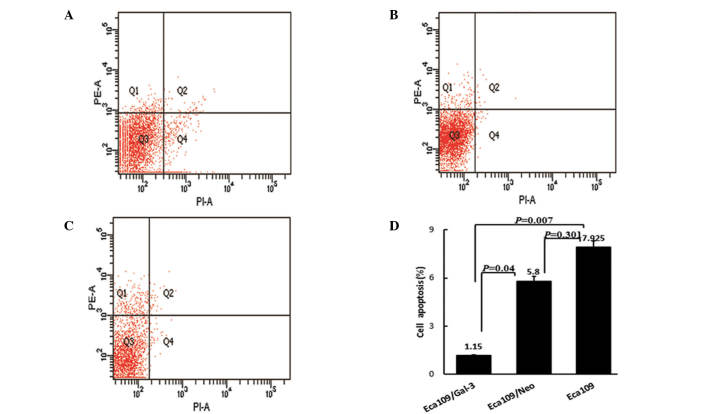
Effect of galectin-3 on Eca-109 human esophageal cancer cell apoptosis. (A) Transfected group. (B) Non-transfected group. (C) Negative control group. (D) Quantification of apoptosis. Compared with Eca-109 cells and Eca-109/Neo cells, Eca-109 cells transfected with galectin-3 (Gal-3) exhibited significantly reduced apoptosis (P=0.007 and P=0.04, respectively). No significant differences were observed between the Eca-109 and Eca-109/Neo cells (P=0.301).

The effect of galectin-3 overexpression on the apoptosis of Eca-109 cells was determined by flow cytometric analysis with Annexin V/PE and 7-AAD double-staining. Compared with Eca-109 (7.9±4.4%) and Eca109/Neo cells (5.8±1.69%), the number of apoptotic cells was significantly reduced in the Eca-109/Gal-3 group (1.2±0.26%; P=0.007 and P=0.04, respectively). No significant differences were detected between the Eca-109 and Eca-109/Neo groups (P=0.301; Fig. 3). These results revealed that galectin-3 overexpression inhibited apoptosis in Eca-109 cells.

### Effect of galectin-3 on the migration and invasion capacity of Eca-109 cells

The migration capacity of Eca-109/Gal-3 cells (55.4±3.9), was significantly increased compared with that of either the Eca-109 or the Eca109/Neo cells (P<0.05), while no significant differences were observed between the migration capacities of the Eca-109 and Eca-109/Neo cells (30.6±1.5 and 29±2.6 respectively, P>0.05).

**Figure 4:**
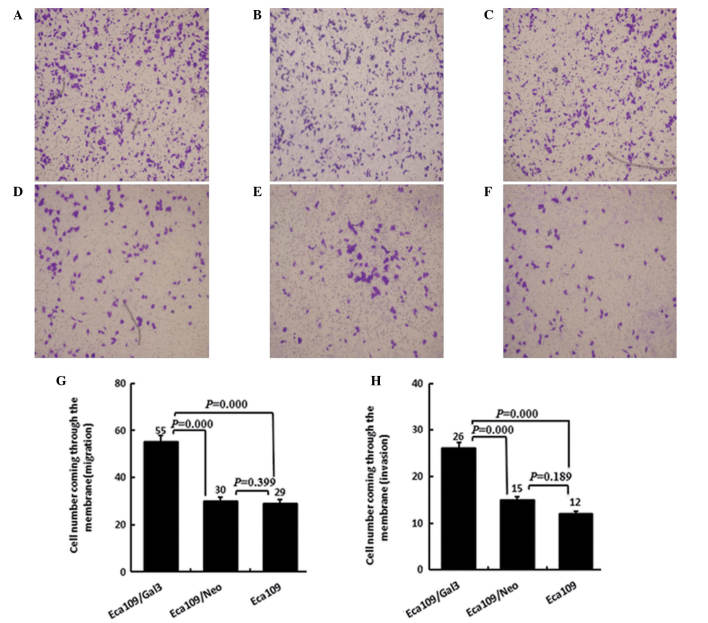
Migration and invasion capacities of the Eca-109, Eca-109/Gal-3, and Eca-109/Neo human esophageal cancer cell groups. Magnification, ×4.2. Migration of (A) Eca-109/Gal-3, (B) Eca-109 cells and (C) Eca-109/Neo cells through the membrane. Invasion of (D) Eca-109/Gal-3, (E) Eca-109/Gal-3 and (F) Eca-109/Neo cells through the membrane. (G) A significantly greater number of Eca-109/Gal-3 cells migrated through the membrane than either Eca-109 or Eca-109/Neo cells (P<0.05). No significant differences were identified between Eca-109 and Eca-109/Neo cells (P=0.399). (H) A significantly greater number of Eca-109/Gal-3 cells migrated through the membrane than either Eca-109 or Eca-109/Neo cells (P<0.05). No significant differences were identified between Eca-109 and Eca-109/Neo cells (P=0.189).

Invasion is an important step in the movement of tumor cells across the extracellular membrane (ECM) in tumor metastasis. Therefore, ECMatrix served as a reconstituted basement membrane matrix protein in invasion assays. Compared with Eca-109 and Eca-109/Neo cells, Eca-109/Gal-3 cells exhibited significantly greater invasiveness across the ECMatrix (26.4±3.2, P<0.05). No significant differences was detected between the Eca-109 and Eca-109/Neo groups (14.8±2.6 and 12.4±2.3 respectively; P>0.05; Fig. 4).

## Discussion

Galectin-3, which is widely expressed in normal and tumor cells, is associated with cell growth, adhesion, differentiation and death, and has been observed to be expressed in colon cancer, gastric cancer and other malignancies (4,8–13). However, to the best of our knowledge, the galectin-3 expression levels in esophageal cancer have not been reported thus far.

Galectin-3 is localized to the nucleus and cytoplasm. Gaudin *et al* (15) reported galectin-3 to be either exclusively cytoplasmic, predominantly nuclear or distributed between the two compartments, depending on cell type and specific experimental conditions. The nuclear versus cytoplasmic distribution of galectin-3 in different cell types may reflect the presence or absence of a potent nuclear export signal or a compartment-specific anchor in the interacting partner. In the present study, a 31-kDa protein band was detected in non-transfected Eca-109 esophageal cancer cells, revealing that galectin-3 is endogenously expressed. This was confirmed using RT-PCR analysis of galectin-3 mRNA expression levels. However, further investigation of primary esophageal cancer tissue is required to determine whether this was an artifact of the Eca-109 esophageal cancer cell line used.

Metastasis is a fatal complication of malignancy. Tumor cell metastasis from the primary to secondary sites is associated with changes in cell adhesion, invasion and migration that allow the survival of metastatic cells in the circulation, and the formation of new vessels. Experimental and clinical studies have revealed that the galectin-3 expressed in tumor cells is important at different stages of tumorigenesis, including malignant cell transformation, invasion and metastasis (6,7,16). O’Driscoll *et al* (17) confirmed that galectin-3 overexpression in a lung cancer cell line significantly enhanced cell motility and invasiveness *in vitro*, indicating that endogenous galectin-3 regulates cancer cell migration.

Integrins have been observed to be expressed in numerous cell types, and exert critical roles in inflammation, apoptosis, proliferation, tumor cell migration and metastasis (18). Notably, galectin-3 has been reported to regulate tumor cell metastasis and invasion by means of activating or expressing integrins (19). Therefore, the effects of galectin-3 on cell adhesion may be mediated by galectin-3 binding to integrins.

Increasing evidence has demonstrated that angiogenesis is essential for tumor growth and metastasis, and is an important factor in cancer progression. Markowska *et al* (20) reported that galectin-3 exhibited angiogenic activity *in vitro*, suggesting that it modulates vascular endothelial growth factor- and basic fibroblast growth factor-mediated angiogenesis through binding of the carbohydrate recognition domain.

In the present study, galectin-3 overexpression was found to be associated with enhanced migration and invasion capacity in Eca-109 cells. This phenomenon indicates that galectin-3 promotes esophageal cancer cell migration and invasion, although confirmation in galectin-3 gene-silenced Eca-109 esophageal cancer cells is required.

The most extensively investigated function of galectin-3 is the regulation of apoptosis, which is dependent on the subcellular localization of galectin-3. The cytoplasmic expression of galectin-3 is antiapoptotic (19). Following exposure of cells to apoptotic stimuli, interaction with proteins, such as the Ca^2+^- and phospholipid-binding synexin, is essential for the translocation of galectin-3 to the perinuclear membrane to inhibit changes in the mitochondrial membrane potential thuspreventing apoptosis (21). Therefore, the antiapoptotic activity of galectin-3 may also be mediated by interaction with other apoptotic regulators that function in the mitochondria.

The regulatory effects of galectin-3 on apoptosis have been shown to be dependent on subcellular localization (22). In the present study, Annexin V/7-AAD double-staining was used to detect the effect of galectin-3 on apoptosis. The results revealed that the apoptotic rate was significantly lower in Eca-109/Gal-3 cells compared with non-transfected Eca-109 cells, suggesting that as galectin-3 expression in Eca-109 cells is antiapoptotic, it must be cytoplasmic. The antiapoptotic function of galectin-3 has been documented in a series of studies. For instance, overexpression of galectin-3 in lung cancer cells was found to be associated with increased resistance to apoptosis compared with that of non-transfected control cells (19). Furthermore, Yu *et al* (21) demonstrated that BT549 human breast cells overexpressing galectin-3 were more resistant to the apoptosis induced by cisplatin, nitricoxide, radiation and anoikis than non-transfected cells. However, in the present study, only the effect of overexpressed galectin-3 on the behavior of human esophageal cancer Eca-109 cells was observed; the effect of a galectin-3-silencing gene on apoptosis in Eca-109 cancer cells has not yet been observed, although this may be analyzed in the future.

In the present study, galectin-3 expression in Eca-109 esophageal cancer cells was confirmed and galectin-3 was implicated as a positive regulator of growth, migration and invasion, and antiapoptotic effector. However, galectin-3 expression in esophageal cancer tissue remains to be confirmed. An improved understanding of the role of galectin-3 in esophageal cancer may provide a novel strategy for the diagnosis and prognosis of esophageal cancer, and the development of novel therapeutic regimens.

## References

[1] Jemal A, Siegel R, Ward E (2008). Cancer statistics, 2008. CA Cancer J Clin.

[2] Barondes SH, Castronovo V, Cooper DW (1994). Galectins: a family of animal β-galactoside-binding lectins. Cell.

[3] Leffler H, Carlsson S, Hedlund M, Qian Y, Poirier F (2004). Introduction to galectins. Glycoconj J.

[4] Lotan R, Ito H, Yasui W (1994). Expression of a 31-kDa lactoside-binding lectin in normal human gastric mucosa and in primary and metastatic gastric carcinomas. Int J Cancer.

[5] Liu FT, Hsu DK, Zuberi RI (1995). Expression and function of galectin-3, a beta-galactoside binding lectin, in human monocytes and macrophages. Am J Pathol.

[6] Takenaka Y, Fukumori T, Raz A (2004). Galectin-3 and metastasis. Glycoconj J.

[7] Nakahara S, Oka N, Raz A (2005). On the role of galectin-3 in cancer apoptosis. Apoptosis.

[8] Schoeppner HL, Raz A, Ho SB, Bresalier RS (1995). Expression of an endogenous galactose-binding lectin correlates with neoplastic progression in the colon. Cancer.

[9] Canesin G, Gonzalez-Peramato P, Palou J (2010). Galectin-3 expression is associated with bladder cancer progression and clinical outcome. Tumour Biol.

[10] Sakaki M, Fukumori T, Fukawa T (2010). Clinical significance of galectin-3 in clear cell renal cell carcinoma. J Med Invest.

[11] Knapp JS, Lokeshwar SD, Vogel U (2013). Galectin-3 expression in prostate cancer and benign prostate tissues: correlation with biochemical recurrence. World J Urol.

[12] Braeuer RR, Shoshan E, Kamiya T, Bar-Eli M (2012). The sweet and bitter sides of galectins in melanoma progression. Pigment Cell Melanoma Res.

[13] Honjo Y, Nangia-Makker P, Inohara H, Raz A (2001). Downregulation of galectin-3 suppresses tumorigenicity of human breast carcinoma cells. Clin Cancer Res.

[14] Pfaffl MW (2001). A new mathematical model for relative quantification in real-time RT-PCR. Nucleic Acids Res.

[15] Gaudin JC, Mehul B, Hughes RC (2000). Nuclear localisation of wild type and mutant galectin-3 in transfected cells. Biol Cell.

[16] Yang RY, Rabinovich GA, Liu FT (2008). Galectins: structure, function and therapeutic potential. Expert Rev Mol Med.

[17] O’Driscoll L, Linehan R, Liang YH (2002). Galectin-3 expression alters adhesion, motility and invasion in a lung cell line (DLKP), in vitro. Anticancer Res.

[18] Hood JD, Cheresh DA (2002). Role of integrins in cell invasion and migration. Nat Rev Cancer.

[19] Liu FT, Rabinovich GA (2005). Galectins as modulators of tumour progression. Nat Rev Cancer.

[20] Markowska AI, Liu FT, Panjwani N (2010). Galectin-3 is an important mediator of VEGF- and bFGF-mediated angiogenic response. J Exp Med.

[21] Yu F, Finley RL, Raz A, Kim HR (2002). Galectin-3 translocates to the perinuclear membranes and inhibits cytochrome c release from the mitochondria. A role for synexin in galectin-3 translocation. J Biol Chem.

[22] Haudek KC, Spronk KJ, Voss PG (1800). Dynamics of galectin-3 in the nucleus and cytoplasm. Biochim Biophys Acta.

